# The safety and efficacy of acupuncture for epididymitis protocol for a systematic review

**DOI:** 10.1097/MD.0000000000013934

**Published:** 2019-01-04

**Authors:** Jisheng Wang, Liang Han, Binghao Bao, Xudong Yu, Kaige Zhang, Hengheng Dai, Xiao Li, Bin Wang, Haisong Li

**Affiliations:** a Graduate School of Beijing University of Chinese Medicine; b Department of Andrology, Dongzhimen Hospital; c Department of Andrology, Fangshan Hospital, Beijing University of Chinese Medicine, Fangshan District; d Department of Encephalopathy, Dongzhimen Hospital, Beijing, China.

**Keywords:** acupuncture, epididymitis, protocol, systematic review

## Abstract

**Background:**

Epididymitis is a common disease in non-specific infections of the male reproductive system. According to the clinical incidence of acute epididymitis and chronic epididymitis, which is more common in chronic epididymitis. There are many clinical trials confirmed that acupuncture treatment can relieve pain and improve symptoms of epididymitis to some extent. In this systematic review, we aim to evaluate the effectiveness and safety of acupuncture for epididymitis.

**Methods and analysis:**

We will search for PubMed, Cochrane Library, AMED, EMbase, WorldSciNet; Nature, Science online and China Journal Full-text Database (CNKI), China Biomedical Literature CD-ROM Database (CBM), and related randomized controlled trials included in the China Resources Database. The time is limited from the construction of the library to November 2018. We will use the criteria provided by Cochrane 5.1.0 for quality assessment and risk assessment of the included studies, and use the Revman 5.3 and Stata13.0 software for meta-analysis of the effectiveness, recurrence rate, and symptom scores of epididymitis.

**Ethics and dissemination:**

This systematic review will evaluate the efficacy and safety of acupuncture for epididymitis. Because all of the data used in this systematic review and meta-analysis has been published, this review does not require ethical approval. Furthermore, all data will be analyzed anonymously during the review process trial.

**Registration number:**

PROSPERO CRD42018111348.

## 1 Introduction

Epididymitis, which is a common disease in non-specific infections of the male reproductive system, can be found in male of all ages, but most of them are young adults aged 20 to 40, which accounting for 70% of all the patients who suffer from it.^[1,2]^ It could, according to the clinical incidents, be classified into 2 types which we define them as acute epididymitis and chronic epididymitis, and the chronic type is more common. The main clinical manifestations contain referred pain in hypogastrium and groin, enlarged scrotum, increased pain when standing or walking.^[3]^ When it is attacking in acute phase, the clinical features such as local redness and pain, sudden rise in temperature and leukocytosis will be manifested. Besides, nodules or lumps or turning hard could be detected through physical examination, accompanied with mild or moderate tenderness, and the vasodilatation of vas deferens could also be detected on the pathological side.^[4]^ The cause of epididymitis is complicated and varied, but the pathogenesis of the disease is usually identified as secondary to prostatitis or urinary tract infections. The disease has been seriously affecting patients’ mental health and quality of life.^[5]^ Meanwhile, it could also reduce the production and development of sperm because of the various degree of the inflammation in the epididymis, which resulting in a significant increase in sperm deformity, therefore, the risk of suffering from infertility is increased.^[6–8]^

Currently, definite therapy for epididymitis has not been settled, the use of antibiotics and anti-inflammatory drugs are still the main treatment.^[9]^ But for the recurrent chronic epididymitis, the drugs could not be easily effective because of the susceptibility of nodules.^[10,11]^ Besides, the chronic type could suffer from varicocele easily and the local congestion would be formed naturally, which lessens the local immunity and leads to the lingering disease more easily.^[12,13]^ Aiming at this situation, a few patients accept the therapy of epididymectomy which, however, does not enhance the overall efficacy because of the deficiency of the quality of sexual life.^[14,15]^

Acupuncture, 1 important part of traditional Chinese medicine (TCM), has been widely used in clinical trials in recent years. It has been undoubted that acupuncture has obvious curative effect in reducing chronic pain in the epididymis, reducing scrotal swelling and anti-tissue fibrosis, which have been shown by recent studies.^[16]^ The studies also have shown that acupuncture could achieve the desired results of peripheral analgesia, which mainly by the means of stimulating related acupoints in order to accelerate the production of endogenous opioid peptides in the central nervous system and activate the analgesic system in the body of the patient.^[17,18]^ Besides, it could also achieve anti-inflammatory effects by increasing the levels of β-Ep in inflammatory tissues and serum.^[19,20]^ According to the theory of TCM, we believe that acupuncture could regulate the balance of qi and blood in human body, which ameliorates the function of the human body by stimulating the regulated acupoints. This kind of therapy has been increasingly popular among doctors and patients because of the unique advantages of simplicity, convenience, efficacy, and low cost.

After a preliminary search and analysis of database resources, we found that a rising trend of randomized controlled trials (RCTs) of acupuncture for epididymitis is becoming visible. However, most clinical trials confront with the inferior quality of the studies with small sample size and the insufficiency of evidence-based exploration because of the limitation of the size and number of clinical centers. Therefore, we expect to adopt meta-analysis to evaluate the efficacy and safety of acupuncture in the treatment of epididymitis, in order to provide evidence for its clinical application.

## 2 Methods

This systematic review protocol has been registered on PROSPERO CRD42018111348 (https://www.crd.york.ac.uk/PROSPERO/display_record.php?RecordID=111348). The protocol follows the Cochrane Handbook for Systematic Reviews of Interventions and the preferred reporting items for systematic reviews and meta-analysis protocol (PRISMA-P) statement guidelines. We will describe the changes in our full review if needed.

### 2.1 Inclusion criteria for study selection

#### 2.1.1 Types of studies

We will gather all studies of acupuncture therapy in treating epididymitis which, no matter whether they have been published or not, base on the method of randomized controlled trial (RCT). The language is limited to Chinese and English. Non-RCTs quasi-RCTs, series of case reports and cross research will be excluded.

#### 2.1.2 Types of participants

Male patients who have been diagnosing as epididymitis will be included, which means that there is no limitation in age, regional, national, ethnic, and sources.

#### 2.1.3 Types of interventions

Including the use of acupuncture,electro-acupuncture, fire, plum needle, the massage on the related acupuncture points, acupuncture treatment of epididymitis as experimental interventions. Considering that the theory of pharmaco-acupuncture and point injection belongs to another part of TCM, so they will be considered for exclusion.

##### 2.1.3.1 Control interventions.

As for the control interventions, who accepted virtual acupuncture treatment can be used as a placebo-controlled or did not get any treatment as a blank control would be adopted. However, once they had accepted acupuncture combined medication or other therapy of TCM, the trials will be rejected.

The following treatment comparisons will be investigated:

(1) Acupuncture versus no treatment;
(2) Acupuncture versus placebo/sham acupuncture;
(3) Acupuncture versus drug therapy;
(4) Acupuncture versus other active therapies;
(5) Acupuncture with another active therapy versus the same therapy alone.

#### 2.1.4 Types of outcome measures

##### 2.1.4.1 Primary outcomes

The main criterion is epididymal color Doppler ultrasonography.

##### 2.1.4.2 Secondary outcomes

Secondary evaluation criteria included that whether there was amelioration of the symptoms of patients, tenderness in epididymis, recovery in size and changes in safety indicators (blood routine urine routine liver and kidney function). Meanwhile, whether there were occurrences of adverse reactions or adverse events during the experiment should be paid close attention to, which in order to comprehensively evaluate the clinical efficacy and safety of acupuncture for epididymitis.

### 2.2 Search methods for the identification of studies

#### 2.2.1 Electronic searches.

Database Search: Search PubMed, Cochrane, Library, AMED, EMbase, WorldSciNet; Nature Science online and China National Knowledge Infrastructure (CNKI), China Biology Medicine disc (CBMdisc). The temporal interval is limited from the time that the databases created to November 2018, and the combination of keyword and free word retrieval is adopted. The search terms include “acupuncture”,“electro-acupuncture”,“fire needle”,“skin needle”,“plum blossom needle”,“ epididymitis”, and “sub-sputum”. The search term in the Chinese database is the translation of the above word. The complete PubMed search strategy is summarized in Table 1.

**Table 1:**
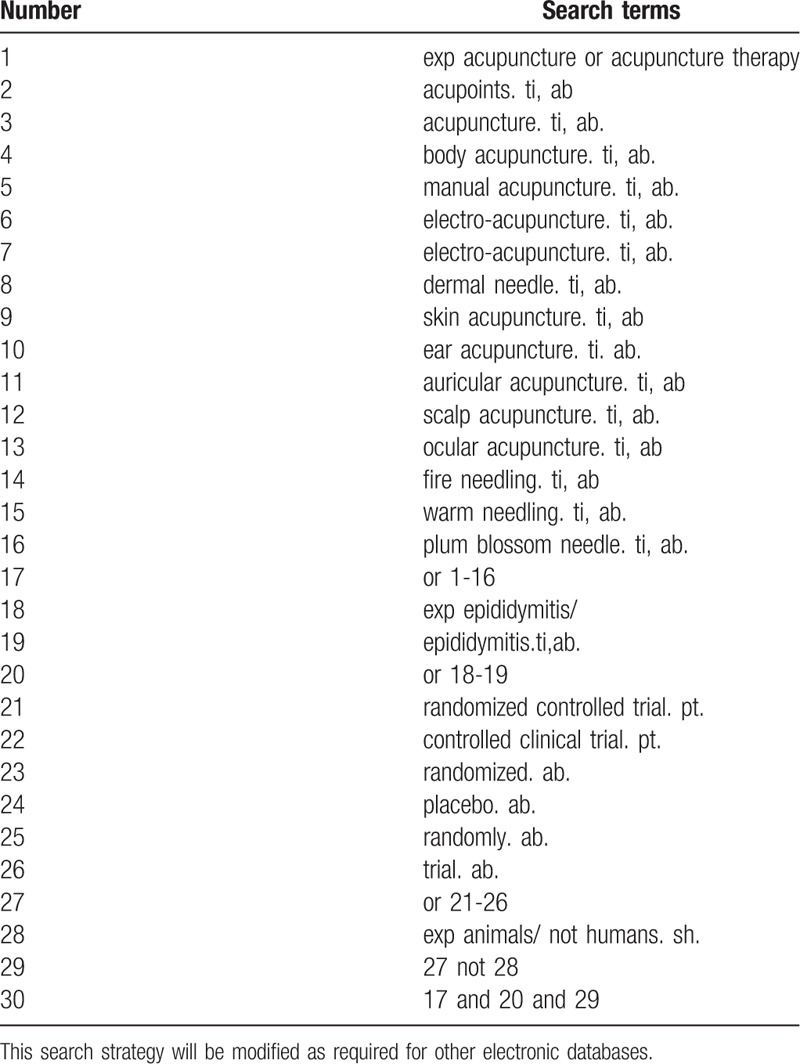
Search strategy used in PubMed database.

#### 2.2.2 Searching other resources

The manual search mainly searched for relevant literatures, earlier than the database above-mentioned, such as “China Rehabilitation Medicine Journal”, “Chinese Acupuncture”, “Chinese Journal of Physical Medicine and Rehabilitation”, “Acupuncture Clinical Journal”, and “Chinese Journal of Urology”.

### 2.3 Data collection and analysis

#### 2.3.1 Study identification

**Figure 1:**
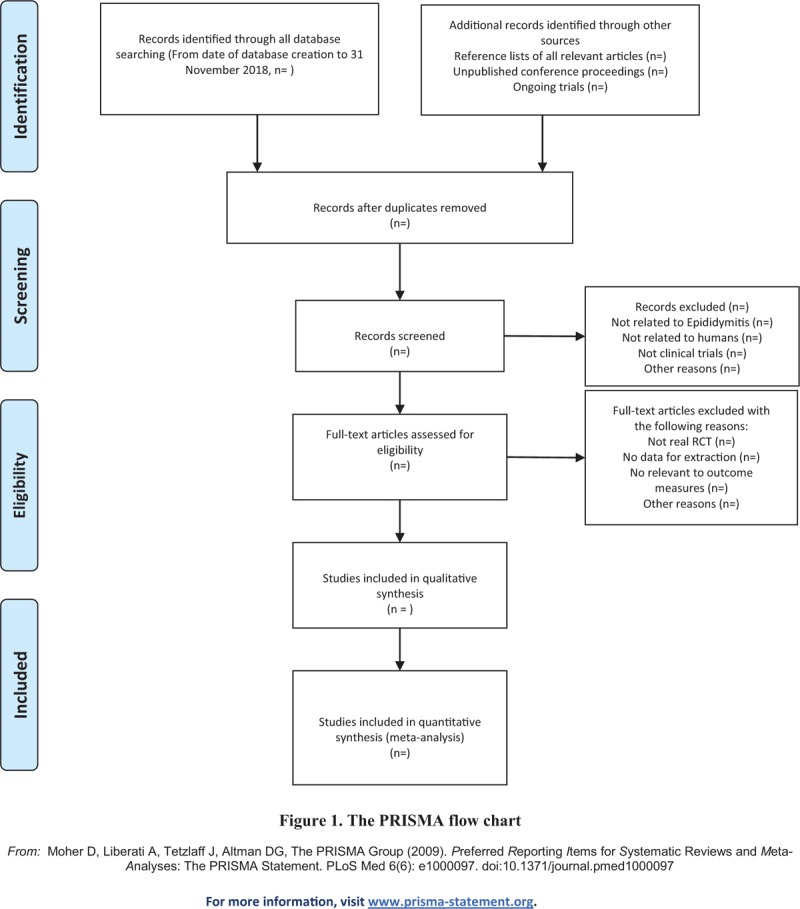
The PRISMA flow chart. PRISMA = preferred reporting items for systematic reviews and meta-analyses.

(1) There are 2 researchers filtering out the literature that clearly do not conform to the study such as meeting minutes dissertations reviews animal experiments and so on, which, after excluding all the retrieved documents from the duplicated literature, adopt the method of reading the title of the literature abstracts, and so on.
(2) The second time of screening the literature: skimming the remaining documents and filtering out unqualified documents such as case reports theoretical discussions and non-conformance of interventions.
(3) The third time of screening the literature: carefully reading the remaining documents and strictly filtering out unqualified documents such as general controlled trials, lacking control group, deficiency of random allocation, incompatible outcome indicator and the appearance of similar data, and so on.
(4) As for the literature that cannot be ensured, it would be confirmed by the discussion of the 2 researchers. And if they cannot reach an agreement, the third-party experts would get involved, which aims at absorbing the appropriate RCTs into the study.
  The primary selection process is shown in a PRISMA flow chart (Fig. 1)

#### 2.3.2 Data extraction and management

The literature data extraction will be completed independently by 2 researchers and the data form uniformly developed by the researcher was filled out. The data extraction content includes the following:

(1) General information: article title, First author, Corresponding Author, time of publication research, evaluation correspondence, contact information.
(2) Research method: design pattern, ample size, random allocation, random hiding, blind method, baseline level.
(3) Participants: patients age, gender, epididymitis diagnostic criteria, severity, ethnicity study, location.
(4) Intervention: acupuncture, Acupuncture point, period of treatment, treatment frequency
(5) Efficacy evaluation: main observation indicators secondary observation indicators safety indicators and number of adverse reactions.
(6) Note: sources of funds, medical ethics audit, important references

#### 2.3.3 Assessment of risk of bias (ROB) in included studies

As for the Literature quality evaluation, we will use the bias risk assessment tool recommended by Cochrane to assess the quality of all included literature and ROB. The assessment includes:

(1) sequence generation;
(2) allocation concealment;
(3) blinding of participants, personnel, and outcome assessors;
(4) incomplete outcome data;
(5) selective outcome reporting;
(6) other sources of bias.

The evaluation above would be independently evaluated by 2 researchers. If there are different opinions, we discuss them. If there are still differences exist, we would consult the third appraiser. Otherwise, we need to consult with the Cochrane Professional Group for solution.

#### 2.3.4 Statistical analysis

The meta-analysis studied in this review will adopt Rev Man5.3 and Stata13.0 statistical software. Heterogeneity test will be used for the inclusion of the study, and random or fixed effect models will be adopted, with *P* <.05 as the test standard. If the heterogeneity between the results is too large, the random effects model (REM), which deduces the source of heterogeneity by sensitivity analysis, will be used for the rest analysis. Second, according to the different type of statistical data, the binary categorical variable will use the odds ratio (OR) and its 95% confidence interval (CI) as the effect analysis index. As for the continuous variable, the standardized mean difference (SMD) and its 95% CI will be used as the effect analysis index. If the outcome measures only provide the means and standards deviation before or after treatment, the Mean_change_ and the SD_change_ are obtained according to the method provided in Cochrane Handbook 5.1.0: 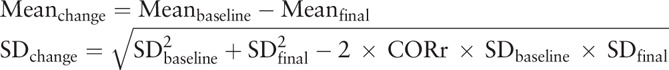


The forest map and funnel plot were drawn and analyzed using Rev Man5.3 software, and the funnel plot was used to analyze potential publication bias. As for the Literature quality evaluation, we will use the bias risk assessment tool recommended by Cochrane to assess the quality of all included literature and ROB. The assessment includes:

1) sequence generation;
2) allocation concealment;
3) blinding of participants, personnel and outcome assessors;
4) incomplete outcome data;
5) selective outcome reporting;
6) other sources of bias.

The evaluation above would be independently evaluated by 2 researchers. If there are different opinions, we discuss them. If there are still differences exist, we would consult the third appraiser. Otherwise, we need to consult with the Cochrane Professional Group for solution.

#### 2.3.5 Assessment of heterogeneity

We will use a X^2^ test to estimate heterogeneity of both the mean difference (MD) and OR. Further analysis can be performed using the I^2^ test. If possible, we will also construct a forest plot for analysis. A random-effect model will be used to interpret the results if heterogeneity is statistically significant, whereas a fixed-effect model will be used if heterogeneity is not statistically significant. We will regard heterogeneity as substantial when I^2^ is greater than 50% or a low *P* value (<.10) is reported for the X^2^ test for heterogeneity.^[16]^

#### 2.3.6 Sensitivity analysis

We will conduct a sensitivity analysis to identify whether the conclusions are robust in the review according to the following criteria: sample size, heterogeneity qualities, and statistical model (random-effects or fixed-effects model).

#### 2.3.7 Publication bias

If a result of a meta-analysis contains more than 10 articles and above, we will use a funnel plot to test the risk of publication bias.

#### 2.3.8 Quality of evidence

The quality of evidence for the main outcomes will also be assessed with the Grading of Recommendations Assessment (GRADE) approach. The evaluation included bias risk; heterogeneity; indirectness; imprecision; publication bias. And each level of evidence will be made “very low,” “low,” erate,” or “high” judgment.

## 3 Discussion

In recent years, the clinical RCT about epididymitis has been increasing continuously, however, it is still unsatisfactory in the diagnosis and therapy of the disease.^[21]^ The clinicians have not reached a consensus on the therapeutic principles and evaluations of the disease, and lacks unified normalized standards. At present, there is no large-scale epidemiological investigation on this disease, and there are few reports in related literature. TCM has a profound theoretical foundation and rich clinical experience in the treatment of chronic epididymitis.^[22,23]^ Acupuncture, which, an essential part of TCM, possesses the characteristics of small side effects and simple and easy operation, has long been used to treat genitourinary diseases such as prostatitis.^[24,25]^ This kind of therapy mainly achieves therapeutic effects by stimulating the body's righteousness and regulating the balance of qi and blood, besides, yin and yang.^[26]^ Although the specific mechanism of acupuncture treatment of epididymitis is not very clear, clinical studies have shown that acupuncture treatment of epididymitis can relieve pain and improve symptoms to some extent.^[16]^ As far as we know, there has not had any comparison of the effectiveness of acupuncture in the treatment of epididymitis.

Therefore, we will use systematic review and meta-analysis to evaluate the efficacy and safety of acupuncture for the treatment of epididymitis. The results of this study can provide a possible ranking for acupuncture treatment of epididymitis. In addition, the quality of evidence for the primary outcome will be assessed using a scoring method. We hope that these results will provide clinicians with the basis for acupuncture treatment of epididymitis and provide the best choice for the treatment of patients. In addition, although this study will conduct a comprehensive search, it will not search for languages other than Chinese and English, which will lead to some bias.

## Author contributions

**Data curation:** Jisheng Wang, Binghao Bao, Kaige Zhang.

**Formal analysis:** Jisheng Wang, Hengheng Dai, Xiao Li.

**Funding acquisition:** Haisong Li.

**Project administration:** Bin Wang.

**Supervision:** Liang Han, Bin Wang.

**Validation:** Haisong Li.

**Writing – original draft:** Jisheng Wang, Xudong Yu, Kaige Zhang.

**Writing – review & editing:** Jisheng Wang.
